# 2013 Update in addiction medicine for the generalist

**DOI:** 10.1186/1940-0640-8-18

**Published:** 2013-11-04

**Authors:** Adam J Gordon, Nicolas Bertholet, Jennifer McNeely, Joanna L Starrels, Jeanette M Tetrault, Alexander Y Walley

**Affiliations:** 1 VA Pittsburgh Healthcare System and University of Pittsburgh, Pittsburgh, PA, USA; 2 Lausanne University Hospital, Lausanne, Switzerland; 3 New York University School of Medicine, New York, NY, USA; 4 Albert Einstein College of Medicine and Montefiore Medical Center, Bronx, NY, USA; 5 Yale University School of Medicine, New Haven, CT, USA; 6 Boston University School of Medicine, Boston, MA, USA

**Keywords:** Primary care, Alcoholism, Addictive behavior, Drug abuse, Substance-related disorders, Screening and brief intervention, Use of pharmacotherapy for addiction treatment in primary care, Integration of addiction treatment and medical care

## Abstract

Increasingly, patients with unhealthy alcohol and other drug use are being seen in primary care and other non-specialty addiction settings. Primary care providers are well positioned to screen, assess, and treat patients with alcohol and other drug use because this use, and substance use disorders, may contribute to a host of medical and mental health harms. We sought to identify and examine important recent advances in addiction medicine in the medical literature that have implications for the care of patients in primary care or other generalist settings. To accomplish this aim, we selected articles in the field of addiction medicine, critically appraised and summarized the manuscripts, and highlighted their implications for generalist practice. During an initial review, we identified articles through an electronic Medline search (limited to human studies and in English) using search terms for alcohol and other drugs of abuse published from January 2010 to January 2012. After this initial review, we searched for other literature in web-based or journal resources for potential articles of interest. From the list of articles identified in these initial reviews, each of the six authors independently selected articles for more intensive review and identified the ones they found to have a potential impact on generalist practice. The identified articles were then ranked by the number of authors who selected each article. Through a consensus process over 4 meetings, the authors reached agreement on the articles with implications for practice for generalist clinicians that warranted inclusion for discussion. The authors then grouped the articles into five categories: 1) screening and brief interventions in outpatient settings, 2) identification and management of substance use among inpatients, 3) medical complications of substance use, 4) use of pharmacotherapy for addiction treatment in primary care and its complications, and 5) integration of addiction treatment and medical care. The authors discuss each selected articles’ merits, limitations, conclusions, and implication to advancing addiction screening, assessment, and treatment of addiction in generalist physician practice environments.

## Introduction

Increasingly, patients with unhealthy alcohol and other drug (AOD) use are being seen in primary care and other nonspecialty addiction settings. Primary care providers are well-positioned to screen, assess, and treat patients with AOD use because these conditions may contribute to a host of medical and mental health harms. An ever-present concern is the length of time that it takes to translate evidence-based AOD screening, assessment, and treatment approaches into real-world settings. Several publications focusing on the integration of AOD care into primary care settings have sought to disseminate the latest evidence to generalist clinicians, including the 2012 Update in Addiction Medicine for the Generalist published in this journal [1-6]. In order to provide a recent update, the authors presented a 2013 Addiction Update for the Generalist at the annual meeting of the Society of General Internal Medicine (SGIM) in the spring of 2013. The following paper describes the literature reviewed during this presentation.

We sought to identify and examine important recent advances in addiction medicine in the medical literature that have implications for the care of patients in primary care or other generalist settings. To accomplish this aim, we selected articles in the field of addiction medicine, critically appraised and summarized the manuscripts, and highlighted their implications for generalist practice, employing methodology used in prior updates [3-6]. During an initial review, we identified articles through an electronic Medline search (limited to human studies and in English) using search terms for alcohol and other drugs of abuse published from January 2010 to January 2012. After this initial review, we searched for other literature in web-based or journal resources (e.g., Alcohol, Other Drugs, and Health: Current Evidence [http://www.bu.edu/aodhealth], the American College of Physicians (ACP) Journal Club, tables of contents of relevant journals) for potential articles of interest. From the list of articles identified in these initial reviews, each of the six authors independently selected articles for more intensive review and identified the ones they found to have a potential impact on generalist practice. The identified articles were then ranked by the number of authors who selected each article.

Through a consensus process in monthly meetings over the course of five months, the authors reached agreement on the articles with implications for practice for generalist clinicians that warranted inclusion. The authors then grouped the articles into five categories: 1) screening and brief intervention in outpatient settings, 2) identification and management of substance use among inpatients, 3) medical complications of substance use, 4) use of pharmacotherapy for addiction treatment in primary care and its complications, and 5) integration of addiction treatment and medical care.

### Screening and brief intervention in outpatient settings

Screening and brief intervention (SBI) is an evidence-based practice model for identifying and addressing unhealthy alcohol use in general health-care settings, and SBI for substance use carries a Grade B recommendation from the United States Preventive Services Task Force (USPSTF) [1]. The SBI approach typically begins with opportunistic screening in general health care settings with a goal of identifying patients with unhealthy substance use who are not seeking substance abuse treatment. Those patients who screen positive are followed up with a brief counseling intervention, which is usually delivered during the same medical visit. Screening, brief intervention, and referral to treatment (SBIRT) programs addressing both drug and alcohol use are widely promoted by health authorities in the US and are supported by insurance billing codes. Nonetheless, SBI is rarely integrated into regular primary care practice [7,8].While there is ample evidence to support the use of SBI for unhealthy or hazardous alcohol use, concerns have been raised about the lack of evidence for SBI for patients with alcohol dependence [9] and for addressing other drug use disorders [7-10].

Evidence supporting alcohol brief interventions in adult primary care settings has largely been derived from efficacy trials [11-14], and important questions remain about how SBI can be implemented within regular medical care and the ‘dose’ of intervention needed for it to be effective. Alcohol SBI research is now moving toward gathering more practice-based evidence, with studies integrating SBI into regular care processes without reliance on research staff. This has exposed the considerable barriers to SBI implementation [4-6,15], including limited time to perform BI and physician knowledge in this area [16-21].

### How effective is alcohol screening and brief intervention when implemented in usual care?

Kaner E, et al. [15], D’Onofrio G, et al. [22] Kaner and colleagues [15] undertook a pragmatic trial of three brief intervention strategies in primary care settings as part of the large multi-center Screening and Intervention Programme for Sensible drinking (SIPS) trial conducted in the UK. Over 3500 patients across 29 primary care practices participated in this cluster randomized trial. Practices were assigned to one of three interventions of increasing levels of intensity: simple feedback plus a patient information leaflet (provided for all interventions); a five-minute structured brief intervention delivered by practice staff; or a brief intervention followed by a 20-minute motivational interviewing session delivered at a follow-up visit by an alcohol counselor. The primary outcome was drinking status as measured by the Alcohol Use Disorders Identification Test (AUDIT).

Hazardous and harmful drinking decreased in all treatment conditions at six and 12 months, but the odds of having a negative AUDIT score did not differ significantly between those who received the pamphlet versus the brief intervention (OR = 0.85) or motivational interviewing (OR = 0.78). Readiness to change appeared to have a dose-related response to the amount of counseling received, but no differences were observed in alcohol-related problems or health-related quality of life. With no treatment-as-usual group, the investigators were unable to evaluate whether the observed changes in drinking outcomes could be attributed to effectiveness of the interventions versus regression toward the mean.

This pragmatic trial illustrated some of the implementation challenges to integrating SBI into primary care settings. Many sites were unable to recruit the initial target of 31 patients per practice. Follow-up rates for the brief lifestyle counseling intervention were relatively low, with just 57% of eligible patients returning for the dedicated counseling visit. The authors concluded that, based on results from this trial, there may be no additional benefit of counseling interventions over simple feedback and written information in addressing unhealthy alcohol use in primary care.

A second study investigating looked at the efficacy of alcohol SBI when delivered by medical staff in the emergency department (ED). Prior research on the impact of brief interventions for unhealthy alcohol use in ED settings has been inconclusive, with a recent meta-analysis showing no significant impact on alcohol consumption but a 50% reduction in alcohol-related injury [23]. This single-site study by D’Onofrio and colleagues [22] examined the effect of a brief intervention conducted by the medical provider (median duration = 7 minutes), with or without a booster call, compared to standard care. Eligibility was restricted to patients with hazardous and harmful drinking (representing 2.8% of all patients screened) and excluded individuals with alcohol dependence. Among the 889 participants, reductions were seen in the mean number of drinks in the past seven days and mean number of binge episodes in the past 28 days at six and 12 months in all treatment conditions. Reduction in drinking was significantly greater in those who received the brief intervention compared with standard care, but there was no additional benefit from the booster call. Rates of driving after drinking were significantly reduced for the brief intervention groups compared with standard care. This study supports the effectiveness of a single brief intervention in the ED, using existing ED staff, for individuals with hazardous or harmful drinking.

### How feasible and effective is implementation of SBI in real-world primary care settings?

Van Beurden I, et al. [24], Hilbink M, et al. [25] In a randomized clinical trial of community-based practices, SBI reduced alcohol-related problems in people with unhealthy alcohol consumption who did not meet criteria for alcohol dependence [26]. While a number of studies have examined strategies to engage providers in addressing hazardous alcohol consumption, there are still relatively few examples of practical implementation trials conducted in routine primary care practice.

Two articles reported findings from a large cluster randomized controlled trial of a tailored, multi-component intervention for addressing hazardous drinking. Seventy-seven practices, including 119 general practitioners and 6318 patients, participated in the study. Hazardous drinking was addressed at three levels: the implementation strategy included *professional*-*directed interventions*, such as guideline distribution, reminder desk cards, and educational training sessions for the care teams; *organization*-*directed interventions*, which included the proportion of patients with hazardous/harmful use and dependence, facilitation of referrals to local addiction programs, and outreach to the practice by a trained facilitator; and *patient*-*directed interventions*, e.g., waiting room posters and patient information letters including personalized feedback on their alcohol use. Intervention practices were randomized to receive all three levels of the intervention. The control practices only received patient-directed information letters. The main outcomes were receipt of screening and advice at 12 months measured at the *practice level* and the proportion of hazardous drinking patients who reported reduced use to low risk levels at two years measured at the *individual level*.

Participating practices were primarily small clinics having one (48%) or two (35%) general practitioners. Implementation of SBI was thwarted by low recruitment and staff motivation (e.g., 13 general practitioners missed all trainings, only half of the intervention practices met the minimum requirements for participation). For the *practice*-*level outcomes*, there were no significant improvements in screening or advice-giving at 12 months. For the *individual*-*level outcomes*, 41.6% of patients with hazardous and harmful drinking reduced their consumption to low-risk levels. However, more (47%) individuals in the control group reduced alcohol use than in the intervention group (36%), which was an effect in the opposite direction than hypothesized.

Large-scale implementation of SBI for alcohol in primary care is challenging, and programs that fail to adequately address provider and patient attitudes, system- and practice-level barriers, education, and provider incentives for providing SBI services may not confer the benefits found in smaller or more intensive efficacy studies [12]. Unfortunately, these reports by Van Beurden and Hilbink do not provide analyses of the individual program components, which could have provided important guidance for future programs. While the findings from this study should be considered alongside examples of successful SBI implementation [17,27], more research focused on determining the most effective strategies for successfully integrating, scaling up, and creating sustainable evidence-based SBI for alcohol use in primary care is clearly needed.

### What is the efficacy of screening and brief intervention for drugs other than alcohol?

Humeniuk R, et al. [28] In contrast to the field of alcohol SBI, where research is moving from efficacy to effectiveness trials, there have been few rigorous clinical trials examining the efficacy of SBI for illicit drugs [10]. A study by Humeniuk and colleagues addressed this important research question in a randomized clinical trial examining the efficacy of a single brief intervention linked to the Alcohol, Smoking and Substance Involvement Screening Test (ASSIST) in primary care settings. The ASSIST is a screening and assessment instrument developed by the World Health Organization (WHO) that screens for lifetime and current substance use and queries a range of substance use-related problems, generating a substance-specific risk score that categorizes use as low, moderate, or high risk [29].

In this study, 731 adult patients with moderate risk use of at least one illicit drug were enrolled from diverse outpatient care sites (including community health centers, sexually transmitted disease clinics, and dental clinics) in four countries (Australia, Brazil, India, and the US). Screening and brief interventions were conducted on-site by research staff, and the intervention focused on the substance of greatest concern (i.e., cannabis, cocaine, or opioids). The primary outcomes were change in global and substance-specific ASSIST scores assessed at three months. Based on an intention-to-treat analysis, the pooled results for all study sites showed a significant reduction in the global substance use score: mean effect size was 18% in the brief-intervention group versus 11% in the control condition (p < 0.001). Decreases were seen in cannabis and stimulant use scores in some countries, and opioid use scores decreased in India (the only country with sufficient prevalence to assess this substance class). No significant effect was seen at the US sites on either global substance ASSIST scores or on cannabis or stimulant scores. The authors speculated that lengthier consent procedures used in the US diminished the treatment effect of brief intervention in these sites. Important limitations of this study were the use of research staff to conduct all screening and interventions and the use of change in ASSIST score, which is of unknown clinical significance, as the primary outcome. Overall, this study indicates that SBI may be effective for some drugs and in some settings, but more research is needed on the efficacy of SBI for drugs other than alcohol.

### Implications for practice

Primary care providers should perform SBI for unhealthy alcohol use as recommended by the USPSTF, and expanding these services to ED settings should be considered. Introducing SBI into busy general health care settings continues to pose a challenge, and more research is needed to guide implementation of best practices. An example of an alternative implementation approach is a study in adolescent primary care clinics that used computer-delivered SBI in the waiting room, where it was well-accepted, doubled rates of BI counseling by primary care providers, and significantly reduced alcohol use [30]. The studies reviewed here also suggest that more may not be better when it comes to the dose of brief intervention, and that it can be difficult to motivate patients to return to clinic for additional counseling, even when it is offered in the same location where they receive primary care. Finally, while there may be benefits to combining AOD screening and interventions (and, in many populations, this may be a clinically-sound approach), the efficacy of SBI for reducing unhealthy illicit drug use remains in question.

### Identification and management of substance use among inpatients

Among patients with alcohol and/or drug dependence, medical complications and comorbidity are common [31], and substance use disorders have been shown to lead to high rates of ED visits and inpatient hospitalizations [32,33]. Substance use may negatively impact hospital stay, undermine discharge planning, and lead to readmissions and increased health care use [34]. At the same time, hospitalization also represents a teachable moment and an opportunity to perform interventions and initiate treatment.

### What is the influence of a substance use disorder diagnosis on acute care hospital utilization?

Walley AY, et al. [35] This observational cohort study examined the rate (total number of ED visits and re-hospitalizations per subject) and risk (proportion of subjects with an ED visit or rehospitalization) of acute care hospital utilization within 30 days of hospital discharge. It is a secondary data analysis of Project RED (Re-Engineered Discharge), a randomized trial of reengineered discharge services for adults admitted to Boston Medical Center from January 2006 through October 2007 [36,37]. The outcomes were determined by medical record review for health-care utilization occurring at Boston Medical Center (all subjects) and by phone interview for utilization occurring elsewhere (86% of subjects were contacted). Substance use disorders were identified by discharge diagnostic codes from the International Classification of Diseases, 9th Revision (ICD-9), and subjects were classified as having an alcohol use disorder diagnosis, a drug use disorder diagnosis, or both.

At discharge, 17% of subjects had an identified substance use disorder diagnosis. Subjects with substance use disorders had higher rates of recurrent acute-care hospital utilization than subjects without (0.63 versus 0.32 events per subject at 30 days, p < 0.01) and an increased risk of any recurrent acute care hospital utilization (33% versus 22% at 30 days, p < 0.05). Adjusting for known factors associated with readmission (age, sex, having a primary care physician, depression symptoms, homelessness in the past three months, employment and insurance status, Charlson Comorbidity Index score, and project RED group assignment), the incident rate ratio at 30 days was 1.49 (95% confidence interval [CI], 1.12-1.98) for subjects with substance use disorders compared to those without. The odds ratio for the risk of reutilization was 1.38 (95% CI, 0.88-2.17) for subjects with substance use disorders compared with those without, but this increase was not statistically significant. In a secondary analysis, higher utilization was attributable to those with drug use disorder diagnoses or a combination of drug and alcohol use disorder diagnoses, but not to those exclusively with alcohol use disorder diagnoses.

These findings demonstrate that substance use disorders are common among medical inpatients and warrant consideration by policy makers, insurance companies, and hospitals in developing new strategies intended to reduce rehospitalization among hospital inpatients.

### Can screening for unhealthy alcohol use identify patients at risk for increased postoperative health-care utilization?

Rubinsky A, et al. [38] A previous study showed that the AUDIT-C (an alcohol screening test comprised of the three consumption questions from the AUDIT) can help identify patients at risk for postoperative complications [39]. This cohort study was conducted among 5171 male Veterans Health Administration (VA) patients of at least 21years of age who completed the AUDIT-C on mailed surveys from October 1, 2003, to September 30, 2006, and who were hospitalized in the subsequent year for at least one day for nonemergent noncardiac major operations. The study assessed whether the AUDIT-C could help identify patients at risk for increased postoperative health-care use. The outcomes were postoperative health-care use (hospital length of stay, number of days spent in the intensive care unit [ICU], return to the operating room in the 30 days after surgery, hospital readmission in the 30 days post-discharge) evaluated across four AUDIT-C risk groups: nondrinkers (score of 0) and low-risk (1 to 4), at-risk (5 to 8), and high-risk (9 to 12) drinkers.

More than half of the study sample reported drinking in the past year; 1853 (36%) were low-risk drinkers, 649 (13%) were at-risk drinkers, and 230 (4%) were high-risk drinkers. Compared with the low-risk group, high-risk drinkers had longer postoperative hospital lengths of stay (5.8 days [95%, 5.0-6.7] versus 5.0 days [95% CI, 4.7-5.3]), more ICU days (4.5 [95% CI, 3.2-5.8] versus 2.8 [95% CI, 2.6-3.1]), an increased probability of return to the operating room in the 30 days after surgery (10% [95% CI, 6-13%] versus 5% [95% CI, 4-6%]), but no increased hospital readmission within 30 days post-discharge (7% [95% CI, 4-11%] versus 6% [95% CI, 5-7%]). Analyses were adjusted for age at the time of surgery, race, marital status, service-connected disability, past-year smoking status, surgical procedure category based on Current Procedural Terminology codes (i.e., cardiovascular or thoracic, musculoskeletal, gastrointestinal, genitourinary, or other—a proxy measure of surgical complexity), and days from alcohol screening to surgery. At-risk drinkers did not differ significantly from low-risk drinkers on any measure of postoperative health-care use.

This study demonstrates that a simple screening test for alcohol use can help identify patients at risk for postoperative health care use. Further research is needed to elucidate the contributors of increased risk in this population and to develop effective interventions to mitigate this risk.

### How can substance use be addressed at the hospital to increase continuity of care and transition to addiction treatment?

Pecoraro A, et al. [40] Addressing substance use disorders among inpatients can decrease acute-care utilization and may lead to engaging patients in addiction care. The authors completed a retrospective data evaluation of a pilot program (Project Engage) which was conducted at Wilmington Hospital in Delaware. Its purpose was to identify and facilitate entry of medical and surgical inpatients to substance use disorder treatment after discharge. The program was designed to provide bedside assistance for the clinical team to address substance use disorders. Patients with substance use disorders were identified by clinicians (based on clinical judgment, without systematic screening). Patients received motivational interviewing and facilitated referral to treatment by a patient engagement specialist. Patient engagement specialists were in stable recovery from alcohol and/or drugs for at least two years. They provided one to two motivational-interviewing sessions while the patients were at the hospital to enhance motivation to attend substance use treatment; discussed potential treatment programs when patients were willing to consider substance use treatment; determined whether programs had openings; investigated whether and how treatment could be funded; made appointments; and addressed potential barriers to treatment (e.g., homelessness, clothing, transportation). They called patients within 48 hours of the planned admission or appointment. The outcomes were self-reported rates of substance-use treatment initiation among all patients. Of the 415 patients who participated in Project Engage, 180 (43%) were admitted to a substance-use treatment program. Most (57%) attended outpatient addiction facilities, while 29% were admitted to residential treatment.

These results are promising and suggest that the identification and facilitation of addiction treatment entry are worthwhile. Nevertheless, given the nature of the study and its design, there is a need for additional studies focusing on continuity of care and transition to addiction treatment.

### What is the evidence for smoking cessation interventions among inpatients?

Rigotti N, et al. [41] Numerous studies have assessed the impact of conducting smoking cessation interventions while patients are hospitalized. In this Cochrane systematic review, Rigotti and colleagues determined the effectiveness of interventions for smoking cessation initiated at the hospital for patients who were current smokers or recent quitters. They conducted a systematic review of randomized and quasi-randomized trials of behavioral, pharmacological, or multicomponent interventions started at the hospital to help patients stop smoking.

Interventions were categorized into four groups according to intensity and presence of post-discharge supportive contacts: 1) single contact in hospital lasting 15 minutes or less with no follow-up support, 2) one or more contacts in hospital lasting a total of more than 15 minutes with no follow-up support, 3) any hospital contact plus follow-up for up to one month, and 4) any hospital contact plus follow-up over one month.

Counseling interventions (n = 25) that began during the hospital stay and continued with supportive contacts for at least one month after discharge increased smoking cessation rates following discharge (risk ratio [RR], 1.37; 95% CI, 1.27-1.48) (25 trials). Interventions of lower intensity did not show significant increased smoking cessation rates. Adding nicotine replacement therapy to an intensive counseling intervention increased smoking cessation rates compared with counseling alone (RR 1.54; 95% CI 1.34-1.79) (six trials). Adding varenicline (two trials) or bupropion (three trials) to intensive counseling had no significant effect.

These results indicate that counseling interventions for smoking cessation delivered in a hospital are effective as long as patients are also offered supportive contacts post-discharge, and that nicotine replacement therapy is likely to increase the efficacy of counseling delivered at the hospital.

### Is hospitalization an acceptable moment to perform brief intervention for unhealthy alcohol use?

Broyles LM, et al. [42] The implementation of SBIRT is encouraged in inpatient settings but, questions remain with regard to its acceptability and efficacy. Broyles and colleagues investigated whether nurse-delivered brief intervention would be acceptable to inpatients in a cross-sectional survey of 370 hospitalized medical-surgical patients at a large university-affiliated VA medical center. Factors potentially associated with acceptability were assessed. Patient acceptability was high; most patients agreed/strongly agreed that it would be okay to be asked about their alcohol use or to discuss it with a nurse (95%), and agreed/strongly agreed that if drinking were impacting their health, a nurse should advise them to cut down (94%) or quit (84%) drinking. Patients were less accepting of the offer to be referred to an alcohol treatment program (78% of patients agreed or strongly agreed that it would be okay). Patients were more accepting of SBIRT if they felt able to determine their alcohol risk level, believed that it would be possible to reduce their alcohol-related health risks, and expressed some degree of concern about their own use of alcohol. Approximately 20% of patients reported some degree of personal discomfort with alcohol-related discussions.

These results indicate that patients, even when reporting some degree of discomfort with the topic, find it acceptable for nurses to talk to them about alcohol while they are at the hospital, indicating that SBIRT implementation in hospital may be feasible. Factors associated with SBIRT acceptability in this study tended to suggest that those who are more likely to be in need of an intervention are those who are also less likely to accept it. Furthermore, these results should be considered within the context of the conflicting evidence with respect to alcohol SBIRT efficacy among inpatients [43,44].

### Implications for practice

In summary, there is growing evidence of the importance of substance use as a major determinant of health among inpatients; it can impact readmission, length of stay, and postoperative outcomes. Available screening tools for unhealthy alcohol use can be used to predict postoperative outcomes and hospital stay. Hospitalization and hospital discharge may represent an opportunity to prevent negative consequences of substance use. Further studies focused on coordinated and integrated interventions and treatments for inpatients with substance use are needed.

### Medical complications of substance use

The burden of medical complications of substance use on the population increases with greater use. Here, we focus on the complications of three of the most commonly used substances: marijuana, alcohol, and opioid analgesics. The past decade has seen loosening of marijuana regulation at the state level, including provisions allowing medical marijuana use in 18 states and recreational use in Colorado and Washington, but questions remain about the safety of marijuana use. Alcohol use is widespread, but most studies of alcohol-related complications have evaluated the effects of moderate to heavy alcohol use, and less is known about the impact of light to moderate alcohol consumption, which is more common. In the past decade, as prescription of opioid analgesics has markedly increased. the US has seen substantial increases in non-medical use of opioid medications, the number of diagnosis of opioid use disorders, and overdose deaths. We report on two efforts to reverse this: prescription monitoring programs and abuse-deterrent formulations.

### Does long-term marijuana use cause lung disease?

Pletcher MJ, et al. [45] It is well-established that smoking tobacco causes lung disease, but data about the impact of marijuana use on pulmonary function are limited. This 20-year prospective study of 5115 adults evaluated the long- and short-term effects of smoking marijuana, tobacco, or both on pulmonary function tests. They found that while tobacco use was associated with linear decreases in forced expiratory volume in the first second (FEV1) and in forced vital capacity (FVC), consistent with previous studies, marijuana use had no adverse effect on pulmonary function in individuals who smoked up to 10 “joint-years” during their lifetime (e.g., an average of one joint per day for 10 years, or two joints per day for five years). Light marijuana use was associated with increases in both FEV1 and FVC, but there was a nonsignificant trend towards decreased FEV1 with higher levels of use, indicating a possible negative effect of marijuana use on pulmonary function at high levels of intake.

### Does marijuana use increase the risk of motor vehicle accidents?

Asbridge M, et al. [46] Driving under the influence of marijuana has increased in prevalence, but the evidence about collision risk is inconsistent. This systematic review and meta-analysis of observational epidemiologic studies evaluated the impact of recent marijuana use on the risk of collision resulting in serious or fatal injury. Recent marijuana use was determined by either whole blood toxicology or self-reported use within three hours preceding the collision. In a pooled analysis of 49,411 participants in nine studies, the risk of collision nearly doubled after using marijuana without other substances (OR, 1.92 for any collision [95% CI, 1.35, 2.73], p <0.001; and 2.10 for fatal collision [95% CI, 1.31, 3.36), p = 0.002).

### Does light alcohol use increase the risk of cancer?

Bagnardi V, et al. [47] Moderate and high alcohol consumption increases one’s risk of developing colorectal, breast, laryngeal, liver, esophageal, and oropharyngeal cancer, but little is known about the impact of light alcohol consumption. This study was a meta-analysis of case–control or cohort studies that reported cancer outcomes among individuals with alcohol intake of up to one drink per day (“light drinkers”), compared with individuals with no alcohol consumption. They identified 222 studies with approximately 92,000 light drinkers with cancer and found that light drinking was associated with cancer of the oropharynx (RR, 1.17 [95% CI, 1.06-2.29), female breast (RR, 1.05 [95% CI, 1.02-1.08), and squamous cell carcinoma of the esophagus (RR, 1.30 [95% CI, 1.09-1.56). They found no significant associations of light drinking with colorectal, laryngeal, or liver cancer. These findings highlight that even light alcohol use carries risk.

### Is light alcohol intake protective against heart disease?

Roerecke M, et al. [48] Results of prior studies indicate that low to moderate alcohol consumption might be cardioprotective, but the evidence is limited. In particular, the dose–response relationship is unknown and may differ in women and men. This study was a systematic review and meta-analysis of case–control or cohort studies that reported ischemic heart disease morbidity and mortality, by average alcohol consumption, using lifetime abstainers as the reference group. The authors identified 44 studies, with approximately 960,000 participants. Even with this large amount of data, results were intriguing but not conclusive because of substantial heterogeneity and confounding. They found J-shaped curves for both women and men, indicating that low alcohol use was associated with lower reduced ischemic heart disease mortality, but higher alcohol use was associated with greater ischemic heart disease mortality. For women, up to one drink per day was cardioprotective (RR, 0.84 [95% CI, 0.74-0.96]). For men, three drinks per day was cardioprotective (RR, 0.78 [95% CI, 0.63-0.97]), but surprisingly, one or two drinks was not. This study gives further support to the conclusion that light alcohol may be cardioprotective, but effects differ by sex and further research is needed.

### Are prescription monitoring programs effective at reducing opioid analgesic abuse or overdose?

Reifler L, et al. [49] Over the past decade, states throughout the US have responded to increases in prescription drug abuse and overdose by implementing prescription monitoring programs (PMPs), which variably allow prescribers to access state-run databases of pharmacy fills for controlled substances. Goals of PMPs are to help prescribers identify undisclosed sources of controlled substances, identify patients who are abusing or diverting their prescribed opioids, and ultimately, to reduce the prevalence of opioid abuse and overdose. To date, only limited equivocal data exist about the effectiveness of PMPs in reducing prescription drug abuse or overdose. This ecologic study of states with and without PMPs is one of the first national studies of PMP effectiveness. On a quarterly basis from 2003–2009, states were classified as either having a PMP or not, and then by two measures of opioid analgesic abuse: intentional exposures reported to poison centers and use to get high among individuals entering drug treatment. They found that states with a PMP had a lower quarterly increase in both of these measures, although only the intentional exposures reported to poison centers measure was statistically significant (an increase of 1.91% per quarter in states with no PMP that quarter versus an increase of only 0.2% in states with a PMP, p = 0.036). These findings differ with results by Paulozzi and colleagues [50], who found that PMP presence in a state did not predict opioid overdose mortality.

### What is the relationship between opioids analgesic abuse and heroin abuse? Will efforts to reduce the supply of opioid analgesics lead to an unintended increase in heroin use and potential opioid overdoses?

Unick GJ, et al. [51] Using national data from the Nationwide Inpatient Survey and the US Census, the authors analyzed hospitals’ annual rates of heroin overdose and prescription opioid overdose. They found than an increase in a hospital’s prescription opioid overdose rate predicted subsequent increase in heroin overdose rate, and the reverse was also true. They concluded that use and consequences of these two types of opioids are “intertwined.” Data from the National Survey on Drug Use and Health raise further concern about an increase in heroin use in recent years [52]. Together, these findings may portend greater problems with heroin in the future as national efforts to reduce prescription opioid misuse are implemented and are a call for vigilance and preventative measures.

Cicero TJ, et al. [53] Cicero and colleagues analyzed oxycodone and heroin use among patients entering treatment for opioid dependence quarterly from July 2009 through March 2012 to identify changes before and after release of the abuse-deterrent formulation of long acting oxycodone in August 2010. They found a decrease in the percent of patients using long-acting hydrocodone and an increase in the percent of patients using heroin associated with the new formulation. These findings highlight the importance of considering that removal of one source of opioids is likely to increase another potentially more dangerous one.

### Implications for practice

Generalists are likely to face increasing inquiries from patients seeking guidance about or prescriptions for legalized, medical marijuana, and it is important to keep abreast of emerging science. Recent studies in this review indicate that light marijuana use is unlikely to have adverse effects on pulmonary function, but patients should be cautioned against driving or operating heavy machinery after marijuana use. Although it appears that light alcohol use may reduce cardiovascular risk, the amount of reduction is not known, and patients may be at risk of developing certain cancers even with light use. Therefore, at this time, generalists should not encourage light alcohol intake. The recent increases in opioid analgesic misuse and overdose are very concerning, and generalists should take precautions when prescribing opioids. Clinicians and public health officials should be aware that efforts to reduce the supply of prescription opioids could have a negative consequence of increasing heroin use.

### Use of pharmacotherapy for addiction treatment in primary care

Currently, pharmacotherapies are available to treat alcohol, opioid, and nicotine dependence. Although several other medications are currently under investigation, the three approved medications available to treat alcohol use disorders include disulfiram, an aldehyde dehydrogenase inhibitor; naltrexone, an opioid antagonist which modulates dopamine in the nucleus accumbens; and acamprosate, a GABA agonist/glutamate antagonist. Available pharmacotherapies for opioid use disorders include methadone, a full opioid agonist; buprenorphine, a partial opioid agonist at the mu opioid receptor; and naltrexone, an opioid antagonist. Finally, various nicotine replacement therapies, buproprion (a dopamine reuptake inhibitor originally marketed as an antidepressant) and varenicline (a partial nicotine agonist) are available pharmacotherapeutic agents for smoking cessation. Several recent studies have addressed important clinical questions regarding the use of pharmacotherapy for addiction treatment in primary care.

### Are certain subpopulations of alcohol-dependent patients more likely to respond to naltrexone pharmacotherapy?

Fucito L, et al. [54] Nearly half of alcohol-dependent patients also smoke cigarettes [55], and nicotine dependence is associated with a greater urge to drink, increased risk of relapse after treatment, and heavier drinking upon relapse [56]. Naltrexone is an effective treatment for alcohol dependence [57,58]. This secondary data analysis of the Combined Pharmacotherapies and Behavioral Interventions for Alcohol Dependence (COMBINE) study [59] assessed whether smoking moderated naltrexone’s effect on drinking in alcohol-dependent patients and whether naltrexone had any effect on smoking among alcohol-dependent patients. Of the 1383 patients enrolled in the study, 55% smoked cigarettes, and cigarette smoking was associated with poorer treatment retention and outcomes. Among smokers, those receiving naltrexone had a higher percentage of days abstinent from alcohol (mean = 78 days [+/− SE 0.97] versus mean =72 days [+/− SE 1.8], p = 0.004), and lower number of drinking consequences (mean = 13.6 [+/− SE 1.04] versus mean = 17.5 [+/− SE 1.1], p = 0.01) as measured by the 50-item Drinker Inventory of Consequences Questionnaire. Nonsmokers reported a similar percentage of days abstinent (mean = 74.0 days [+/− SE 2.0] versus 74.6 days [+/− SE 1.8], p = 0.8) and drinking consequences (mean 9.7[ +/− SE 0.9] versus 9.5 [+/− 0.8], p = 0.9) regardless of treatment assignment.

Smoking is prevalent among patients with alcohol dependence and is a predictor of poor treatment retention and outcomes [56]. However, alcohol-dependent smokers receiving naltrexone do benefit from naltrexone with improved alcohol use outcomes [58].

### Is varenicline associated with serious adverse cardiovascular events?

Prochaska J, et al. [60] Varenicline has been found to be an effective agent for smoking cessation and for sustaining abstinence [61]; yet, in a recent study, the rate of cardiovascular serious adverse events was 7% in the varenicline group and 5.7% in the placebo group [62]. This systematic review and meta-analysis investigated the rate of serious treatment-emergent cardiovascular adverse events (defined as occurring during treatment or within 30 days of discontinuation) in all published randomized controlled trials of varenicline for tobacco cessation [60]. Twenty-two trials were included; two studies included patients with active cardiovascular disease, and 11 included patients with a history of cardiovascular disease. The authors explored four different summary measures to assess cardiovascular risk: the Mantel-Haenszel versions of the risk difference, relative risk, odds ratio, and the Petos odds ratio. A summary estimate for risk was also calculated by comparing the four summary statistics at the trial level by grouping the studies by presence versus absence of events and equal versus unequal numbers of events, and then ordering the groups by increasing evidence of a varenicline effect. Rates of treatment-emergent serious cardiovascular adverse events were 0.63% (34 of 5431) in the varenicline groups and 0.47% (18 of 3801) in the placebo groups. Based on the four different summary measures, varenicline treatment was not associated with increases in the risk of serious cardiovascular adverse events. The summary estimate for risk difference was 0.27% (95% CI, 0.10-0.63, p = 0.15), which was neither statistically, nor likely, clinically significant.

Paying attention to bias and differences in study design, the authors of this systematic review and meta-analysis—which included all published trials to date—found no increase in serious treatment-emergent cardiovascular adverse events associated with varenicline use for tobacco cessation. Most trials included patients with current or past cardiovascular disease, and more than one-third of studies did not observe a single serious adverse event.

### Do combined pharmacotherapy and behavioral interventions have efficacy for smoking cessation?

Stead L, et al. [63] Combination pharmacotherapy and behavioral counseling is often recommended in addiction treatment, but the effect size of combined treatment is not known. This systematic review included 41 randomized or quasi-randomized studies evaluating combination treatment approaches for smoking cessation among more than 20,000 participants. One large study, which implemented an intensive intervention that included multiple group sessions, long-term maintenance, recycling contacts, and extended availability of nicotine gum, contributed substantially to study heterogeneity [64]. Of the 40 studies not focusing on such an intensive intervention, evidence suggested a benefit of combination pharmacotherapy (often including nicotine replacement therapy) and behavioral treatment (RR = 1.82 [95% CI, 1.66-2.0]) with moderate statistical heterogeneity (12-40%). Interventions combining pharmacotherapy and behavioral support improved smoking cessation outcomes compared with minimal intervention.

### Is implantable naltrexone efficacious for treating polysubstance abuse?

Tiihonen J, et al. [65] Long-acting naltrexone reduces opioid use in patients with opioid dependence and alcohol use in patients with alcohol dependence, but polydrug use is common, and there is no effective pharmacotherapy for polydrug dependence. The objective of this investigation was to examine the effectiveness of implantable naltrexone in patients with concurrent heroin and amphetamine dependence. This was a trial of 100 outpatients in St. Petersburg, Russia with both heroin and amphetamine dependence who were randomized to receive a 1000 mg naltrexone implant versus placebo for 10 weeks, analyzed via an intent-to-treat approach. Study retention was 52% for patients receiving naltrexone versus 28% for patients receiving placebo (p= 0.01). Thirty-eight percent of patients in the naltrexone group (n = 19) had drug-free urine samples compared with 16% (n = 8) in the placebo group (p = 0.01), with 52% versus 20% having opioid-free urine tests (p< 0.001) and 40% versus 24% having amphetamine-free urine tests (p = 0.09). Despite significant study discontinuation, this study confirms that patients with concurrent opioid and amphetamine dependence receiving long-acting naltrexone have improved treatment retention and decreased heroin use compared with patients receiving placebo. Although long-acting naltrexone may be effective for decreasing amphetamine use in this population, further study is warranted.

### Implications for practice

Safe and effective pharmacotherapies exist for alcohol, opioid, and nicotine dependence. However, the recent literature has addressed important questions regarding the use of these pharmacotherapies in clinical care. Naltrexone is known to be an effective treatment for reducing heavy drinking in alcohol-dependent patients, and this effect has been demonstrated in patients who also smoke. The safety of varenicline for tobacco use has been questioned; however, it is not associated with treatment-emergent cardiovascular adverse events in patients with or without pre-existing cardiovascular disease. Additionally, nicotine dependence responds better to combination pharmacotherapy and brief behavioral counseling over either modality alone. Finally, investigation into a single pharmacotherapeutic agent, namely implantable naltrexone, to treat coexisting heroin and amphetamine dependence confirmed the efficacy of this agent to treat opioid dependence but did not show efficacy for amphetamine use outcomes.

### Integration of addiction treatment and medical care

The Institute of Medicine has called for collaboration and coordination among care providers for patients with combinations of medical, mental health, and substance use disorders [66]. Integration of medical, mental health, and addiction treatment has potential benefits for patients, such as the creation of a “one-stop shop” requiring fewer visits to fewer places for patients and fostering longitudinal relationships with the care team that can increase understanding and trust [67]. For providers, integrated care facilitates daily direct communication and collaboration that results in a team of providers sharing the burden of caring for complicated patients. Several recent studies addressed important clinical questions for the integration of addiction treatment and medical care.

### Does improved quality of chronic disease management among primary care patients with addiction result in improved addiction outcomes?

Kim TW, et al. [68] Health-care reform encourages addiction treatment to shift from acute care delivery that is separate from the rest of health care to a chronic disease model more integrated within health homes that can manage patients with multiple overlapping problems [69]. It is not clear how quality of chronic disease management (CDM) relates to addiction treatment outcomes.

This secondary analysis of data from a primary care-based CDM randomized clinical trial for patients with addictions determined the association of 1) engagement in care and 2) CDM quality with addiction severity and abstinence at follow-up. The primary-care–based CDM team in this intervention included a nurse, social worker, internist, and psychiatrist, all of whom were experienced in the care of patients with substance use disorders. Engagement in care was defined as at least two visits to the CDM clinic team within 30 days of initiating care. Quality of CDM was measured by the Patient Assessment of Chronic Illness Care (PACIC) survey for both CDM clinical care, specifically, and for their overall addiction care. The PACIC is a 20-item instrument where higher scores indicate care consistent with the chronic care model. Engagement in the CDM was not associated with abstinence or addiction severity. Higher self-reported quality of CDM clinical care was associated with lower drug use severity. Higher self-reported quality of overall addiction care was associated with increased odds of abstinence and lower alcohol severity. Perceived quality of CDM care was associated with better addiction outcomes regardless of the source of the CDM care, whereas a visit-based, frequency measure of care engagement was not.

### Can buprenorphine/naloxone be used for patients with pain and addiction?

Fox AD, et al. [70], Pade PA, et al. [71] Pain is common among patient with opioid dependence [72]. Buprenorphine is a partial opioid agonist FDA-approved in sublingual tablet and film form for the treatment of opioid dependence (combined with naloxone to deter intravenous use), and in transdermal and injectable forms as an analgesic. Due to the ceiling effect of buprenorphine’s opioid activity, opioid-dependent patients with chronic pain may not be good candidates for buprenorphine, and guidelines recommend methadone for these patients. Furthermore, primary care providers may be ill-equipped to treat these patients without help from pain or addiction specialists.

Two studies addressed the issue of using buprenorphine/naloxone among chronic pain patients with opioid dependence. First, a prospective cohort study of 82 patients initiating buprenorphine/naloxone for opioid dependence investigated whether chronic pain was associated with retention in care or opioid use at six months [70]. Using the Brief Pain Inventory instrument, 60% of patients reported chronic pain at baseline, and 38% reported persistent pain at follow-up. No significant difference in retention or opioid use was detected at follow-up for patients with pain at baseline or persistent pain at follow-up. For those with pain at baseline, mean pain scores decreased from 7.3 to 6.0 at six months.

Second, a retrospective chart review of 143 veterans with opioid dependence and chronic pain, who were treated with buprenorphine through a primary-care–based co-occurring disorders clinic, investigated treatment retention and pain scores [71]. Only 11% reported heroin as their primary opioid of abuse; the remainder had prescription opioid dependence at baseline. The median daily morphine milligram (mg) equivalent use was 120 mg at baseline. After stabilization on buprenorphine/naloxone, the median daily dose was 16 mg/4 mg. At six months, 65% of patients were retained on buprenorphine/naloxone, and 5% tapered off all opioids. Pain scores improved from 6.4 to 5.6.

Contrary to guidelines advising caution when prescribing buprenorphine to opioid-dependent patients with chronic pain, emerging literature suggests that buprenorphine is effective for improving both addiction and pain in patients suffering from both conditions. This is encouraging news for primary care providers considering transitioning patients who develop opioid analgesic use disorder in the course of managing their chronic pain onto buprenorphine. Opioid-dependent patients with chronic pain treated with buprenorphine/naloxone receive similar benefits as those without chronic pain. Favorable addiction and pain outcomes can be achieved in patients with opioid dependence stabilized on buprenorphine/naloxone. For the primary care provider treating a patient with buprenorphine for opioid use dependence, this study provides preliminary evidence that pain syndromes can be subsequently treated with buprenorphine.

### How best to deliver hepatitis C treatment to people who use drugs?

Dimova RB, et al. [73], Martinez AD, et al. [74], and Stein MR, et al. [75] Patients with either active or prior substance use are often excluded from hepatitis C treatment clinical trials and receive hepatitis C treatment at lower rates than patients without substance use [76]. This systematic review and meta-analysis of hepatitis C treatment trials that included current and former drug users [73] sought to determine factors associated with treatment completion and sustained viral response. Researchers included 36 studies of 2866 patients who used drugs.

Treatment completion among trial participants who used drugs was 83% and sustained viral response was 55%, which are rates similar to what has been found in subjects of other clinical trials. Incorporation of addiction treatment and social support services into the care plan was associated with higher treatment completion. Using a multidisciplinary team to deliver care was associated with higher sustained viral response to treatment rates.

Two case series of innovative hepatitis C treatment models among methadone maintenance patients, which were recently published and not included in this meta-analysis, reinforce these findings. First, Martinez and colleagues [74] describe an integrated multidisciplinary team in their retrospective record review of 401 patients receiving methadone maintenance treatment who were provided hepatitis C management. The team included an internist, addiction medicine physician, and hepatology expert. Over two years, among 24 patients who initiated treatment, 79% completed treatment and 54% achieved a sustained viral response.

Second, Stein and colleagues [75] describe concurrent group hepatitis C treatment among 42 methadone maintenance patients. In concurrent group treatment, groups of patients initiated treatment at the same time and attended weekly provider and peer-led group sessions that included review of side effects, discussion of treatment adherence, administration of interferon injections, a brief physical exam, and a meditation exercise. In this series, the sustained viral response was 42% in a group of patients who had genotype 1, which was similar to clinical trial rates for this genotype and the treatment they received. Together, the meta-analysis and case series demonstrate that HCV treatment for people who use drugs is feasible and facilitated by integration with addiction treatment.

### Consent

This is a review paper and no formal consent was obtained by the authors of this work for any of the material reviewed. Please refer to the published work regarding any written informed consent obtained from the subjects for each published study.

## Competing interests

The authors declare that they have no competing interests.

## Authors’ contributions

AJG, AYW, and JMT originated the concept of the oral presentation and this paper. All authors presented this work at the 2013 annual meeting of the Society of General Internal Medicine in Denver, Colorado. AJG coordinated the process of gathering and reviewing papers and writing of the manuscript. JM authored the section on SBI in outpatient settings, NB the section on identification and management of substance use among inpatients, JLS the section on medical complications of substance use, JMT the section on use of pharmacotherapy for addiction treatment in primary care, AYW the section on integration of addiction treatment and medical care. All authors read and approved the final manuscript.
